# Injectable self-healing hydrogel delivering trispecific engineered exosomes for osteoporotic bone regeneration via activating mitophagy

**DOI:** 10.1016/j.mtbio.2026.103780

**Published:** 2026-10-27

**Authors:** Ning Xu, Chenjun Liu, Jieyuan Zhang, Huiyong Dai, Shengting Wu, Sa Song, Junjie Guan, Xin Qi, Wei Zhang

**Affiliations:** a National Center for Orthopaedics, Shanghai Sixth People's Hospital, Shanghai, 200233, China; b Department of Orthopaedic Surgery, Shanghai Sixth People's Hospital Affiliated to Shanghai Jiao Tong University School of Medicine, Shanghai, 200233, China

**Keywords:** Osteogenesis, iMSC-derived exosomes, Nucleic acid-based therapeutics, Mitochondrial autophagy, Hydrogel

## Abstract

Osteoporotic bone defects are characterized by impaired matrix remodeling, diminished osteogenic potential, and insufficient vascularization, posing major challenges for tissue regeneration. Mesenchymal stem cell-derived exosomes (MSCs-Exos) have shown promise in promoting osteogenesis and angiogenesis, but their clinical translation is limited by rapid clearance and lack of targeted delivery. Here, we report the development of an injectable, self-healing SK/HA@A hydrogel for sustained delivery of trispecific RNA-engineered exosomes (Tri-Exos) targeting IL31, CELA1, and PCOLCE2. Tri-Exos were engineered via electroporation and rigorously characterized for morphology, size, marker expression, cellular uptake, and functional regulation of target genes in BMSCs. The hydrogel exhibited rapid gelation, robust mechanical properties, dynamic self-healing, and sustained release of Tri-Exos over 30 days. In vitro, SK/HA@A@EVs significantly enhanced BMSC osteogenic differentiation, matrix mineralization, and HUVEC angiogenic responses, while simultaneously activating mitophagy-mediated mitochondrial regulation and autophagy-associated survival pathways, which are critical for maintaining cellular homeostasis under osteoporotic conditions. In a rat femoral defect model, hydrogel treatment improved bone volume, mineral density, and vascularization, as confirmed by micro-CT, histological analyses, and gene expression profiling. Collectively, these results demonstrate that the SK/HA@A@EVs platform orchestrates osteoporotic bone regeneration through synergistic modulation of osteogenesis, angiogenesis, and mitophagy-driven cellular survival pathways. This study provides a translational strategy integrating engineered exosomes with functional hydrogels for targeted, localized therapy of osteoporotic bone defects.

## Highlights

• Exosome-hydrogel system co-delivering siIL31, siCELA1, and mPCOLCE2 is designed to modulate the osteoporotic microenvironment.
• Injectable, self-healing SK/HA@A@EVs show CD63-associated release signals over 30 days and promote single-dose vascularized bone regeneration.
• SK/HA@A@EVs supports osteogenic and angiogenic responses alongside changes in mitophagy- and apoptosis-associated markers.

## Graphical abstract

An injectable, self-healing SK/HA@A@EVs hydrogel enables sustained release of Tri-Exos carrying IL31 siRNA, CELA1 siRNA, and PCOLCE2 mRNA. The released exosomes regulate mitophagy and apoptosis-related pathways in BMSCs, promoting osteogenesis and angiogenesis. This localized delivery system enhances vascularized bone regeneration and improves bone volume and mineral density in osteoporotic defects.

**Figure:**
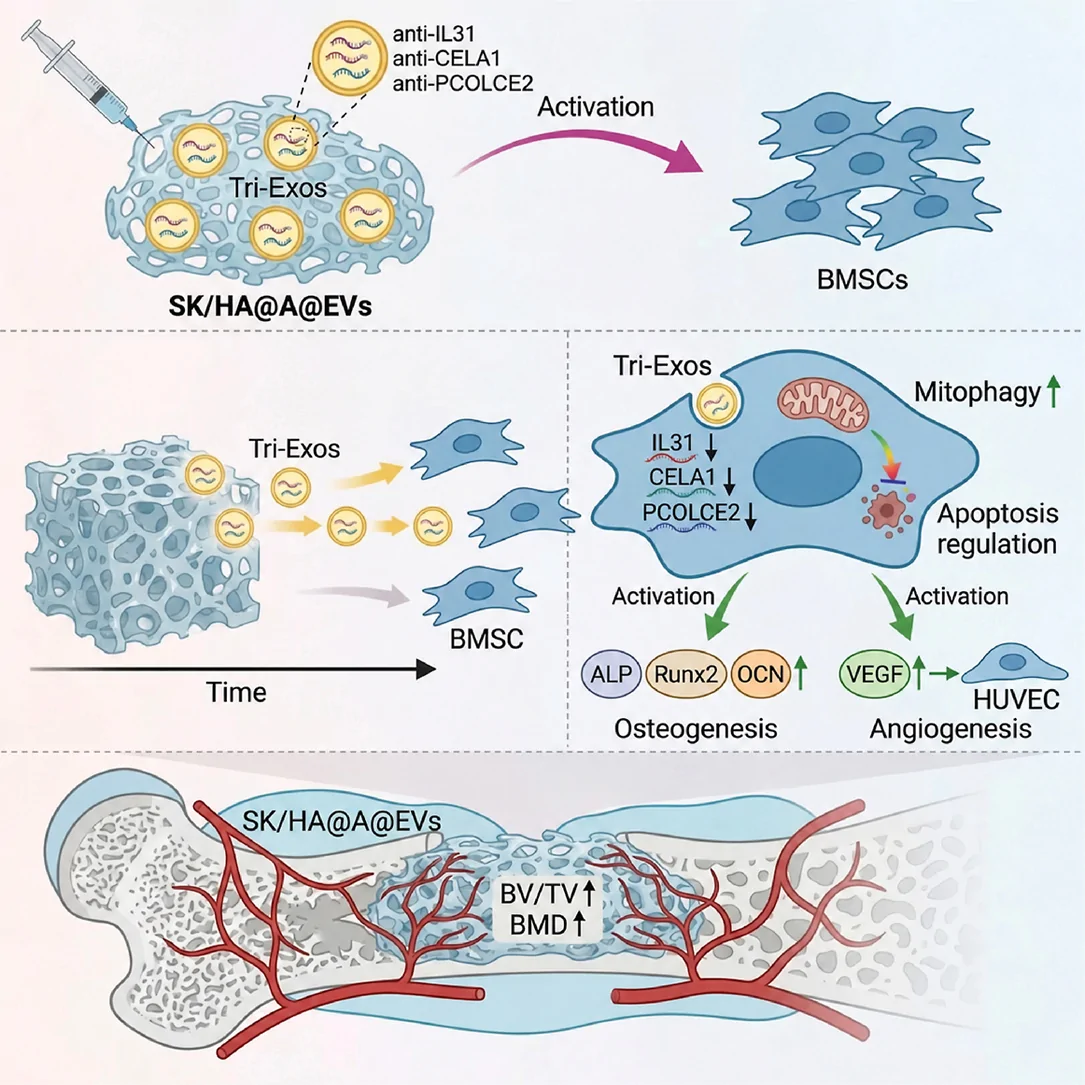

## 1 Introduction

The increasing proportion of elderly individuals has substantially amplified the global burden of skeletal disorders, particularly those arising from osteoporosis, diabetes, and traumatic fractures [1], [2]. These defects result from trauma, infection, tumors, or pathological conditions that disrupt bone homeostasis, impairing the delicate balance between osteogenesis and resorption, and consequently elevating fracture risk [3], [4]. Apoptotic and autophagic pathways further contribute to skeletal pathologies and represent potential therapeutic targets, as dysregulated autophagy can compromise osteoblast and bone marrow mesenchymal stem cell (BMSC) function [5]. Patients often experience delayed fracture healing, especially in poorly vascularized regions, with nonunion rates reaching 15-20%, largely due to BMSC dysfunction and deterioration of the osteogenic microenvironment [6], [7], [8]. Although autologous grafts and synthetic scaffolds can facilitate regeneration, their clinical application is limited by donor scarcity and potential immune rejection [9]. BMSCs hold promise for bone repair through multilineage differentiation, paracrine signaling, and autophagy-mediated cellular homeostasis; however, their regenerative efficacy is hindered by pathological microenvironments and age-related functional decline [10], [11], [12], [13], [14], [15]. Advanced bioinformatics and organoid systems may enhance therapeutic strategies by modeling disease progression and optimizing regenerative interventions [10], [11], [12].

Exosomal vesicles derived from induced pluripotent mesenchymal stem cells (iMSC-Exos, 50-150 nm) have garnered considerable attention due to their bioactive cargo, including proteins, RNAs, and lipids, which facilitate intercellular communication, modulate autophagy, and support tissue regeneration. Compared with direct BMSC transplantation, iMSC-Exos offer superior biocompatibility, reduced immunogenicity, and enhanced stability under pathological conditions [16], [17], [18], [19], [20], [21], [22], [23], [24], [25]. Nevertheless, native iMSC-Exos lack disease-specific therapeutic targeting; therefore, elucidating the molecular mechanisms underlying impaired regenerative capacity in osteoporotic microenvironments—including dysregulated autophagy—and identifying precise molecular targets remain critical.

Identifying disease-associated molecular targets may guide the development of targeted therapies for osteoporosis. Small interfering RNA (siRNA) and messenger RNA (mRNA) offer promising approaches for regulating target gene expression and improving bone regeneration. Small interfering RNA (siRNA) has emerged as a promising next-generation therapeutic modality. With growing understanding of endogenous RNA interference mechanisms, siRNAs are being developed as innovative nucleic acid-based drugs for a variety of diseases, including cancer [26]. Similarly, mRNA-based gene therapies have attracted attention as effective alternatives to recombinant protein treatments for enhancing bone matrix remodeling [27]. However, the in vivo application of siRNA and mRNA is constrained by inefficient cellular delivery. Exosomes can transport siRNAs and mRNAs between cells, modulating target gene expression in recipient cells, and their abundant presence in body fluids underscores their potential as delivery vectors. Electroporation has been employed to load exosomes with RNA cargo efficiently, using pulsed electrical fields to stimulate vesicle production. Compared with conventional transfection methods, this approach enhances exosome yield and cargo loading efficiency, effectively overcoming inherent limitations in extracellular vesicle production and loading [28].

Despite their therapeutic potential, the clinical application of exosomes is hindered by rapid clearance and limited retention at target sites. Hydrogel carriers offer a solution by providing local retention, although they often lack sufficient mechanical strength for bone repair. In this study, sodium alginate (SA) was incorporated as a pore-forming agent to fabricate SK/HA hydrogels with enhanced porosity and controlled release kinetics. Integration of bioactive nanomaterials, such as mesoporous bioactive glass nanoparticles (MBGN), into the hydrogel network has been shown to improve mechanical properties, osteogenic potential, and sustained exosome release [29], [30], [31].

To address the clinical challenges in bone defect repair, we first loaded IL31 siRNA, CELA1 siRNA, and PCOLCE2 mRNA into iMSC-Exos via electroporation and validated the therapeutic efficacy of these functionalized exosomes (Tri-Exos) through in vitro cellular assays. Following confirmation of Tri-Exos efficacy, we engineered a composite hydrogel incorporating Tri-Exos (SK/HA@A@EVs) to stimulate osteogenic differentiation, modulate autophagy, and achieve sustained therapeutic delivery (Scheme 1). The resulting inorganic-organic hybrid hydrogel (SK/HA@A) exhibits excellent injectability, mechanical properties, controlled release, and osteogenic induction potential. This work provides a novel therapeutic strategy for clinical bone defect repair and contributes to advancements in regenerative medicine.

**Scheme 1:**
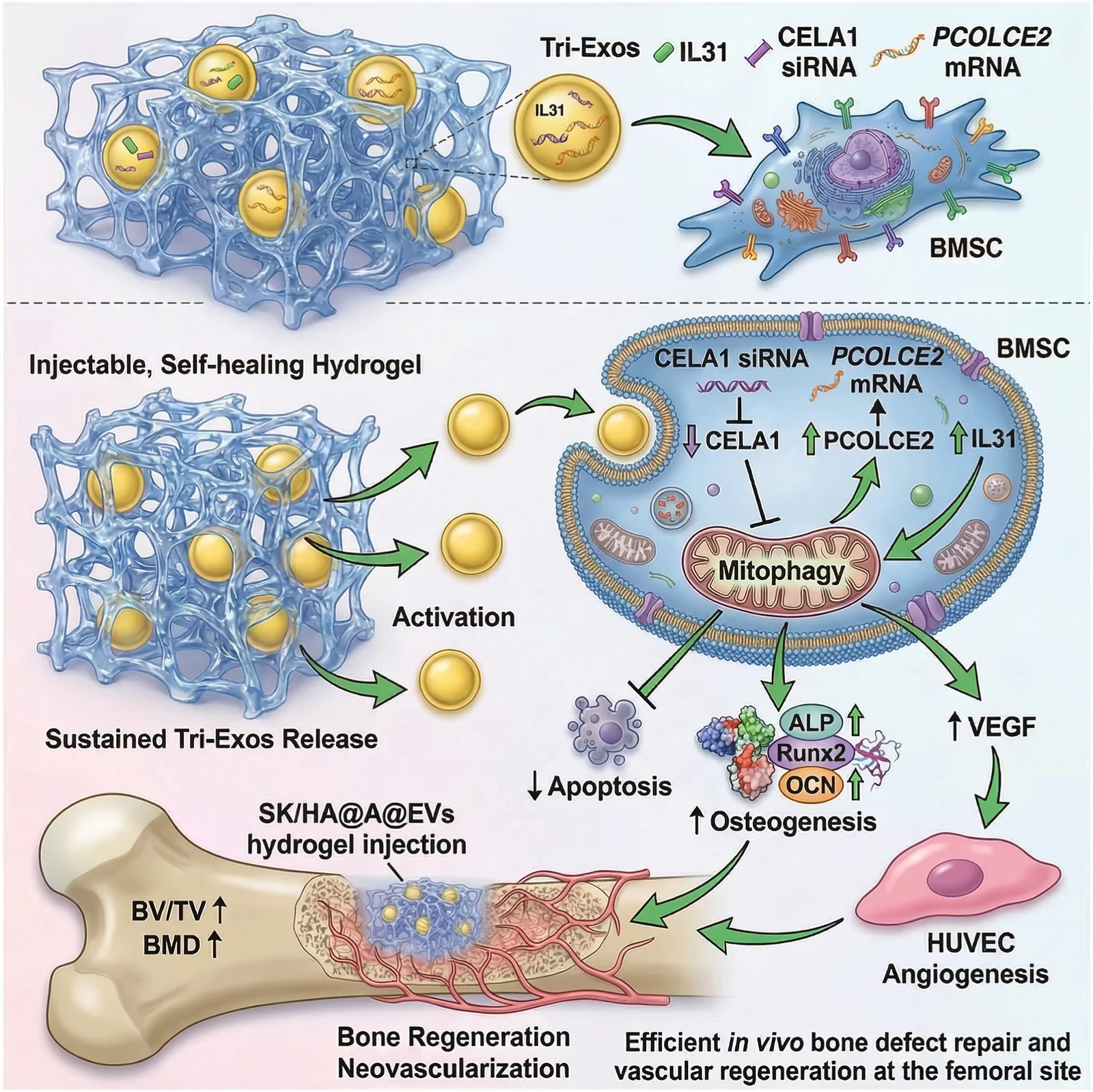
**Mechanistic illustration of SK/HA@A@EVs hydrogel-mediated osteoporotic bone regeneration via Tri-Exos delivery.** The schematic depicts the injectable, self-healing SK/HA@A@EVs hydrogel incorporating trispecific RNA-engineered exosomes (Tri-Exos) targeting IL31, CELA1, and PCOLCE2. Tri-Exos are released from the hydrogel over time and internalized by bone marrow mesenchymal stem cells (BMSCs), where they activate mitophagy and anti-apoptotic signaling, promoting osteogenic differentiation and angiogenic responses.

## 2 Methods

### 2.1 Mendelian randomization (MR) analysis

A bidirectional Mendelian randomization investigation was conducted employing genome-wide association study (GWAS) summary-level data to examine causal relationships between osteoporosis-associated proteins and pathological susceptibility. Genetic instruments achieving genome-wide significance (P < 5 × 10^−8^) for target exposures were utilized, and linkage disequilibrium clumping (r^2^ < 0.01 across 10,000 kb) was implemented to ensure instrumental variable independence. Causal effects were evaluated through multiple analytical approaches: the inverse variance weighted (IVW) method delivered meta-analytic effect estimates under valid instrument assumptions, whereas the weighted median estimator ensured robustness against invalid instruments, and MR-Egger regression identified directional pleiotropy through nonzero intercept evaluation. Effect magnitudes were quantified as odds ratios or beta coefficients with 95% confidence intervals, establishing statistical significance at P < 0.05. All analytical procedures were performed using the TwoSampleMR R package. Bibliometric analysis effectively delineated the contemporary landscape and evolutionary trajectories within the domain, exemplified by computer-aided drug design investigations, thereby establishing strategic frameworks for guiding future bone defect research [32].

### 2.2 Sensitivity analyses

Sensitivity analyses were performed to assess the robustness of the Mendelian randomization (MR) estimates. Cochran's Q tests assessed heterogeneity across variant-specific estimates, MR–Egger intercept tests examined evidence of directional horizontal pleiotropy, and leave-one-out analyses evaluated the influence of individual variants. Both inverse-variance-weighted and MR–Egger analyses were considered when interpreting heterogeneity.

### 2.3 Colocalization analyses

Bayesian colocalization analysis was performed with the coloc R package to evaluate the extent to which osteoporosis GWAS and expression/protein quantitative trait loci (eQTL/pQTL) may share causal variants at selected loci. Colocalization evidence was summarized using posterior probabilities (including PP.H3 and PP.H4), and regional association patterns were visualized using locuscomparer, with variants colored by linkage disequilibrium (r^2^). The MR and sensitivity analyses used the TwoSampleMR and MendelianRandomization R packages [33].

### 2.4 GO and KEGG enrichment analysis

Gene Ontology (GO) and Kyoto Encyclopedia of Genes and Genomes (KEGG) enrichment analyses were conducted with the clusterProfiler R package, employing hypergeometric tests to evaluate differentially expressed genes against a whole-genome background for identifying overrepresented GO terms across the biological process, cellular component, and molecular function categories alongside significantly enriched KEGG pathways, using Benjamini–Hochberg (BH) correction to constrain the false discovery rate to ≤ 0.05. Concurrently, KEGG pathway enrichment was used to map the DEGs to metabolic and signaling pathways and statistically identify disease-relevant mechanisms. These integrated analyses were used to decipher the functional implications of gene expression alterations, elucidating key biological processes and therapeutic targets.

### 2.5 Preparation of Tri-Exos loaded with IL31 siRNA, CELA1 siRNA and PCOLCE2 mRNA

Human induced pluripotent stem cells (iPSCs) were differentiated into mesenchymal stem cells (iMSCs) following standardized protocols and cultured in serum-free StemPro MSC SFM (Gibco) at 37 °C/5% CO_2_ until reaching 80–90% confluency, after which they were incubated for 48 h in exosome-depleted serum-free medium to increase exosome secretion. The conditioned medium was sequentially centrifuged at 300×g (10 min), 2000 × g (20 min), and 10,000 × g (30 min) to remove debris and then ultracentrifuged at 100,000 × g for 2 h to pellet the exosomes. The pellets were resuspended in phosphate-buffered saline (PBS), and all steps performed at 4 °C. To facilitate the successful encapsulation of extracellular vesicles with IL31 siRNA (abbexa), CELA1 siRNA (abbexa), and PCOLCE2 mRNA (abbexa), specific loading parameters were established. A measured quantity of exosomes (20 μg) was placed in a 2-mL sterile tube, followed by the addition of nucleic acid components (0.2 nmol each at a concentration of 100 μM). The solution volume was adjusted to 320 μL using phosphate-buffered saline and allowed to equilibrate for 10 min. This preparation was then transferred into specialized electroporation buffer (Invitrogen) using precision pipettes. Electrical pulse treatment (0.5 kV, 10 ms, 5 cycles) was conducted with Neon transfection equipment (Invitrogen MPK5000). After electroporation, unincorporated genetic material was removed through ultracentrifugation (Beckman Optima XPN-100). The conditioned medium was sequentially centrifuged at 300 g (10 min), 2000 g (20 min), and 10,000 g (30 min) at 4 °C to remove cellular debris. The supernatant was ultracentrifuged at 100,000 g for 1.5 h to pellet exosomes. Purified exosomes were resuspended in PBS, filtered through a 0.22 μm membrane, and ultracentrifuged again (100,000 g, 1.5 h).

Tri-Exos were characterized using transmission electron microscopy (TEM, JEM-1010, JEOL, Japan), and Western blotting [34], [35], [36].Tri-Exos were labeled with PKH26 red fluorescent dye (Sigma-Aldrich) according to the manufacturer's instructions for cellular uptake.

### 2.6 Synthesis of oxidized HA

Formaldehyde-functionalized hyaluronic acid (OHA) was obtained according to a previously described method. Briefly, 2.5 g of HA was dissolved in 250 mL of pure water and stirred at room temperature overnight. Then, 20.8 mL of NaIO_4_ solution was added in the dark and allowed to react for 150 min. Subsequently, 3.5 mL of ethylene glycol was added to terminate the reaction, and the mixture was transferred to a 12-kDa dialysis bag and purified with pure water for three days. The dialysis water was replaced twice daily, and finally, the liquid was freeze-dried to obtain the OHA product, which was stored at −20 °C for future use.

### 2.7 Synthesis and characterization of MBGN and AMBGN

The composition of Mesoporous Bioactive Glass Nanoparticles (MBGN) consisted of 80% SiO_2_, 16% CaO, and 4% P_2_O_5_ synthesized according to established protocols [37]. This procedure involved blending tetraethyl orthosilicate, calcium nitrate tetrahydrate, and triethyl phosphate (all sourced from Shanghai Macklin) within Tris-HCl buffer at pH 8.0, utilizing CTAB (Sigma Aldrich, USA) as the templating agent. Continuous stirring at 60 °C for 24 h preceded washing with ethanol/deionized water and drying, culminating in MBGN powder production via calcination at 500 °C. In accordance with prior protocols, MBGN was aminated using APTES (3-aminopropyltriethoxysilane, Sigma Aldrich, USA), after which 0.3 g of MBGN dispersed in 100 mL of hexane was added to 5 mL of APTES [38]. This mixture was stirred at 60 °C for 24 h prior to repeated washing with ethanol/deionized water, yielding AMBGN for subsequent experiments. X-ray photoelectron spectroscopy (XPS) survey scans and high-resolution N 1s scans of MBGN and APTES-modified MBGN (AMBGN) were used to examine surface nitrogen incorporation.

### 2.8 Preparation and characterization of the SK/HA@A hydrogel system

Methacrylated silk fibroin protein (20 wt%) was mixed with 1% AMBGN and sonicated for 30 min. Then, the sample was quickly mixed with a 5% oxidized hyaluronic acid solution containing 10 mg/mL LAP at a volume ratio of 1:1 and crosslinked under 405-nm blue light to achieve completely solidified SK/HA@A. The rheological behavior of the hydrogel was characterized on a HAAKE RheoStress 6000 system (Thermo Scientific) to observe its self-healing performance. The rheological properties of the hydrogel during solidification were characterized by periodically changing the oscillation amplitude (1%, 500%) and scanning the oscillation time by placing 200 μL of freshly mixed hydrogel on a testing instrument for curing. The internal microstructure of the hydrogel was observed after freeze-drying using SEM (Hitachi, S-4800, Japan) at a voltage of 15 kV.

To conduct compression experiments, cylindrical hydrogel samples with a diameter of 10 mm and a height of 10 mm were prepared. To compare the mechanical properties of samples with different crosslinking durations, a Shimadzu AG-2000A compression tester was used for testing. The loading rate was 5 mm per minute, and the slope of the stress-strain curve during the initial 5% deformation stage was used as the compressive modulus. Cyclic compression tests were performed on SK/HA, SK/HA@M, and SK/HA@A hydrogels using the compression-testing setup described above. The samples were subjected to 20 consecutive compression cycles up to a maximum strain of 20%. In addition, lap shear tests were conducted on gels from different groups to evaluate their adhesion properties.

To examine EV release, SK/HA@A@EVs hydrogels were immersed in PBS. Equal amounts of the supernatant were collected at different times in PBS. Then, a CD63 ELISA kit was used to test the collected samples (ELR-Cd63, RayBiotech).

### 2.9 Rat bone marrow-derived mesenchymal stem cell isolation and culture procedures

Male Sprague-Dawley rats weighing 250–300 g served as experimental subjects, with bone marrow mesenchymal stem cells (BMSCs) undergoing aseptic isolation from bilateral femurs and humeri; marrow suspensions were prepared in α-MEM (Gibco, Invitrogen, Carlsbad, USA) supplemented with 10% fetal bovine serum, 100 IU/mL penicillin, and 100 μg/mL streptomycin and then cultured in dishes at 37 °C with 5% CO_2_. Nonadherent cells were discarded after 48 h, and adherent cells were continuously cultured through medium changes. Upon reaching 80% confluence, the cells were passaged via trypsin digestion. The use of third-passage (P3) BMSCs for all subsequent assays, including proliferation, osteogenic differentiation, and mitophagy-related analyses, ensured experimental reproducibility.

### 2.10 Cell proliferation assay

BMSCs or HUVECs were seeded at 5×10^4^ cells per well in 24-well plates and allowed to adhere. Cells were treated with different experimental groups, including control, hydrogel only, and SK/HA@A@EVs (200 mg/well). Cell proliferation was measured at 24, 72, and 120 h. Cell Counting Kit-8 (CCK-8, Dojindo) was used: 10 μL of CCK-8 reagent was added to each well, incubated at 37 °C for 2 h, and absorbance at 450 nm was measured using a microplate reader. For the exosome comparison, BMSCs were assigned to Control, Exos, and Tri-Exos groups. Cell viability was assessed on days 1, 3, and 5 using the procedure described above. Each treatment condition had at least five replicates, and experiments were independently repeated three times. OD values were used to assess proliferation rates, and data were analyzed using GraphPad Prism software.

### 2.11 Alkaline phosphatase activity assay and alizarin red staining

MSCs were plated in triplicate across three experimental groups within 6-well plates for osteogenic differentiation assessment under the designated treatments. Cells were cultured for 14 days in osteogenic induction medium, and alkaline phosphatase (ALP) activity was quantified per the manufacturer's protocol (Tiandz, Shanghai, China). Following a 21-day treatment period, matrix mineralization evaluation was conducted via alizarin red staining (Sigma, St. Louis, MO, USA), which entailed culture medium removal, two PBS washes, 15 min of fixation in 4% paraformaldehyde, and subsequent rinsing with PBS prior to staining. After 20 min of staining with 2% alizarin red solution at room temperature, phase-contrast microscopy was used to observe bone mineralization and calcium deposition.

### 2.12 Quantitative real-time polymerase chain reaction (RT-qPCR)

The proliferative effect of the SK/HA@A@EVs system on BMSCs was assessed through RT-qPCR, with three biological replicates per condition. On day 3 posttreatment, total RNA was extracted from all three experimental groups using TRIzol reagent, followed by cDNA synthesis with a PrimeScript RT reagent kit (Takara Bio, Shiga, Japan) per the manufacturer's guidelines. Amplification was performed with SYBR Green PCR Master Mix (Takara Bio, Shiga, Japan) using β-actin for normalization. Proliferation-associated target genes were measured via standardized RT-qPCR protocols, and all analyses were executed in triplicate to guarantee accuracy and reproducibility.

### 2.13 Rat femoral defect model experimental design

To investigate the role of the SK/HA@A@EVs system in bone repair, a distal femoral defect model was established in 36 male Sprague-Dawley rats (12 weeks old, 250-300 g). All procedures adhered to ethical guidelines and were performed under sterile conditions. Anesthesia was induced via intraperitoneal injection of 4% chloral hydrate. A longitudinal incision was made to expose the distal femur while preserving surrounding soft tissue. A full-thickness defect (2.5 mm diameter, 3.5 mm depth) was created using a dental drill, avoiding damage to the knee joint and surrounding vasculature. Rats were randomized into three groups (n = 12/group): control group (Blank), SK/HA@A group), and SK/HA@A@EVs group. Each hydrogel contained 200 μg of iMSC-Exos to maintain bioactivity. Wounds were closed layer by layer, followed by intraperitoneal gentamicin (40,000 units/day) for 3 days as infection prophylaxis.

### 2.14 Micro-CT analysis

Twelve weeks post-implantation, femoral specimens were collected for defect evaluation using a micro-CT system (mCT-80, Scanco Medical) at 80 kV, 240 ms exposure time, and 18 μm slice thickness. Three-dimensional reconstructions were generated using CT-Vol software, and bone mineral density (BMD) and bone volume fraction (BV/TV) were quantified. Six specimens per group were randomly selected for new bone formation analysis.

### 2.15 Microvascular perfusion test

We used Microfil (Flowtech) to perform microvascular perfusion after surgery to assess angiogenesis in the bone defect area. The silicone-based contrast agent Microfil was injected into the blood vessels of the rats to make the blood vessels stand out. Upon the completion of perfusion, the rats were euthanized, and the vascular structures were meticulously examined by micro-CT scanning. The scanned pictures were further processed to produce three-dimensional reconstructions of the vascular networks in the defect region. The CT-Vox program facilitated a quantitative examination of the vascular area and vessel count. This allowed comprehensive examination of angiogenesis in the reparative bone tissue by providing a complete picture of the structure of the blood vessels and made it possible to precisely measure how experimental treatments affected the small blood vessels.

### 2.16 Histological and immunohistochemical analysis

At postoperative weeks 2, 4, and 6, rats were intraperitoneally injected with tetracycline hydrochloride (25 mg/kg), alizarin red (30 mg/kg), and calcein (20 mg/kg), respectively, to assess dynamic bone mineralization. Laser scanning confocal microscopy (CLSM, Leica) was used to capture fluorescence signals, where yellow, red, and green corresponded to tetracycline (TE), alizarin red (AL), and calcein (CA) labeling, chronologically marking osteogenic phases. Fluorescently labeled mineralized regions were quantified using ImageJ software.

### 2.17 Statistical analysis

Statistical analysis was performed with SPSS 22.0 software, and the results are presented as the mean ± standard deviation. Data normality was evaluated using the Kolmogorov-Smirnov test, whereas Levene's test was used to verify variance homogeneity. Normally distributed data were analyzed by one-way ANOVA for intergroup comparisons, whereas nonnormally distributed data were analyzed by nonparametric tests. Statistical significance was defined as a p value less than 0.05.

## 3 Results

### 3.1 Construction and functional validation of trispecific RNA-engineered exosomes

To provide a molecularly informed rationale for the design of functional exos, we performed integrative multi-omic analyses of osteoporosis (OP)-related samples, including differential proteomics, Mendelian randomization, and pathway enrichment (Figs. S1–S4). These analyses identified IL31 and CELA1 as risk factors and PCOLCE2 as a protective factor, highlighting key targets involved in bone resorption, extracellular matrix degradation, and collagen maturation (Fig. S3). Upregulated proteins were enriched in proinflammatory signaling and immune–metabolic pathways, whereas downregulated proteins were associated with cell cycle regulation and reduced proliferative capacity. Network analyses further revealed coordinated interactions among inflammatory, mitotic, and osteogenic pathways, indicating hierarchical regulatory mechanisms that contribute to OP pathophysiology. Collectively, these findings established a robust molecular basis for loading iMSC-derived exosomes with these targets, guiding the subsequent preparation and functional characterization of Tri-Exos for targeted modulation of osteoporosis-associated genes.

To establish a molecularly guided platform for osteoporosis therapy, extracellular vesicles (EVs) were isolated from the conditioned medium of induced pluripotent stem cell-derived mesenchymal stem cells (iMSCs) and functionalized with IL31 siRNA, CELA1 siRNA, and PCOLCE2 mRNA via electroporation (Fig. 1A). Prior bioinformatic analyses—including differential proteomic profiling, Mendelian randomization, and colocalization mapping—identified IL31 and CELA1 as pro-osteoporotic factors and PCOLCE2 as a protective gene, providing the rationale for cargo selection in Tri-Exos design. These analyses established the theoretical foundation for the exosome-based intervention in bone metabolic dysregulation.

**Fig. 1:**
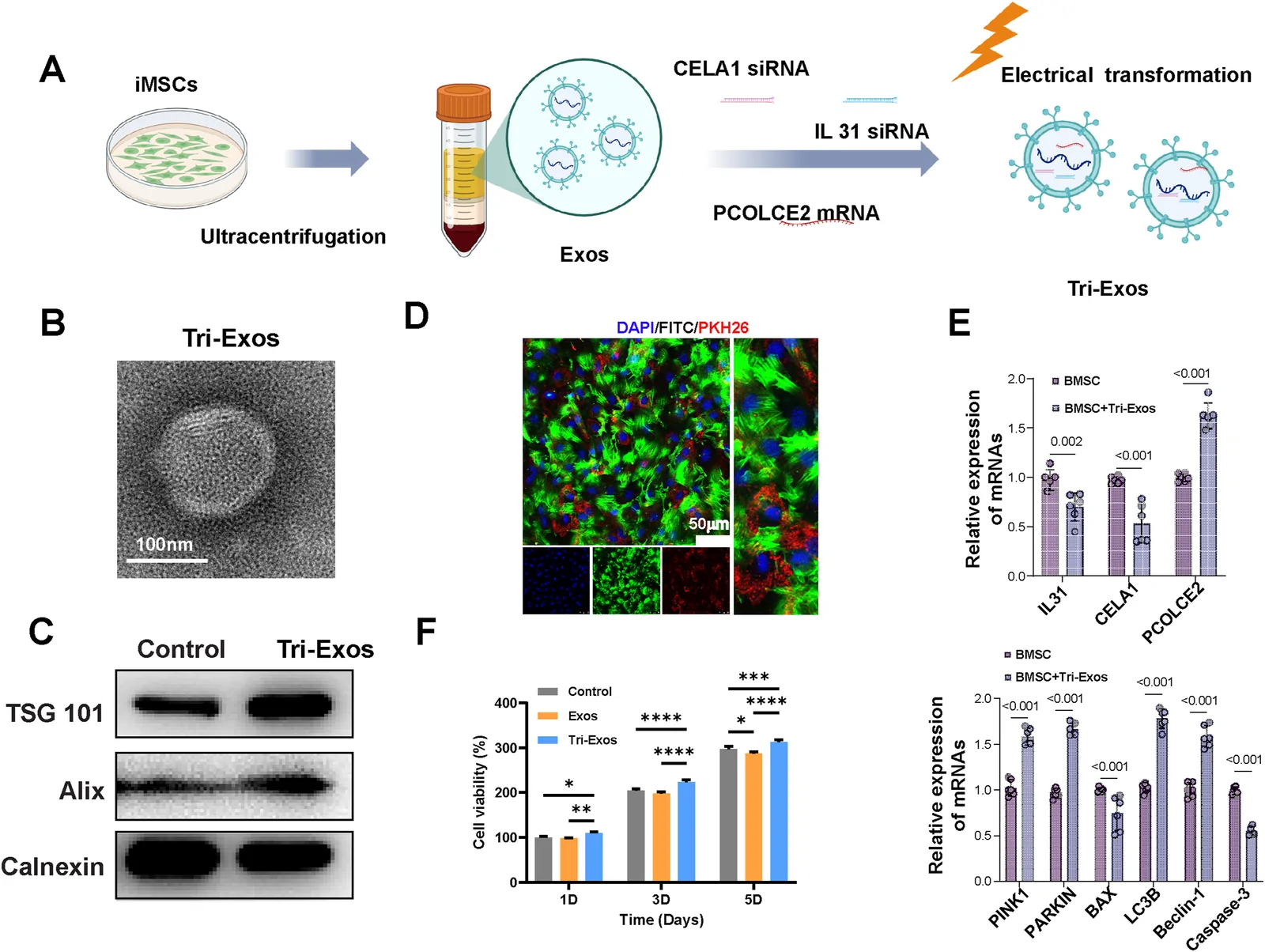
**Construction and functional validation of trispecific RNA-engineered exosomes.**(A) Schematic of Tri-Exos engineering, loading IL31, CELA1, and PCOLCE2 RNAs into MSC-derived exosomes. (B) TEM images of Tri-Exos showing intact spherical morphology and uniform size. (C) Western blot confirming expression of canonical exosome markers (TSG101,Alix and Calnexin) and absence of cellular contaminants.(D) Uptake of exosomes in BMSCs incubated with Tri-Exos. (E) RT-qPCR validation of Tri-Exos-treated BMSCs revealed significantly decreased expression of the risk genes IL31 and CELA1 and upregulation of the protective gene PCOLCE2, suggesting targeted modulation by the biomaterial system. Analysis of the expression of autophagy-related markers in the BMSCs treated with Tri-Exos revealed upregulated expression of PINK1, PARKIN, LC3B, and Beclin-1 and downregulated expression of Caspase-3 and the BAX, indicating enhanced mitophagy and cell survival. (F) The CCK-8 assay was used to detect the proliferation of BMSCs after different treatments. (For interpretation of the references to color in this figure legend, the reader is referred to the Web version of this article.)

After electroporation, Tri-Exos retained a characteristic spherical morphology with diameters between 50 and 200 nm, as confirmed by transmission electron microscopy (TEM) (Fig. 1B), demonstrating structural integrity. Western blot analysis confirmed robust expression of canonical exosomal markers, including Alix and TSG101, while calnexin expression was negligible, indicating minimal contamination from cellular organelles (Fig. 1C). Confocal microscopy demonstrated efficient uptake of Tri-Exos by BMSCs, validating their cellular delivery capability (Fig. 1D).

Functional evaluation revealed that Tri-Exos effectively downregulated IL31 and CELA1 while upregulating PCOLCE2 in recipient BMSCs, establishing a molecular environment conducive to bone regeneration (Fig. 1E). Concurrently, autophagy related markers, including PINK1, PARKIN, LC3B, and Beclin-1 were upregulated, whereas Caspase-3 and BAX expression decreased, indicating activation of mitophagy along with inhibition of apoptosis (Fig. 1E). These findings suggest that Tri-Exos can coordinate multiple cellular pathways to enhance cellular homeostasis. We further compared the effects of Exos and Tri-Exos on BMSC viability. Viability increased over time in all three groups. Tri-Exos showed significantly higher viability than Exos and the Control group on days 1, 3, and 5 (Fig. 1F). These findings suggest that Tri-Exos improve the growth-related response of BMSCs compared with Exos.

### 3.2 Preparation and physicochemical characterization of the SK/HA@A@EVs composite hydrogel system

To construct a robust and bioactive hydrogel scaffold, HA was first oxidized to generate aldehyde-functionalized hyaluronic acid (OHA), which can react with the amino groups of SK and aminated mesoporous bioactive glass nanoparticles (AMBGN) through Schiff base formation (Fig. 2A). TEM imaging confirmed that both MBGN and AMBGN maintained a uniform nanoscale morphology, while elemental mapping verified the presence of silicon and calcium in both nanoparticles and successful surface amination indicated by nitrogen signals in AMBGN (Fig. 2B). Dynamic light scattering measurements showed an increase in particle size after amination, consistent with successful surface functionalization (Fig. 2C). Zeta potential analysis further confirmed the surface charge modification, with AMBGN exhibiting a positive charge compared to the negative charge of MBGN (Fig. 2D). Fig. 2E shown the FTIR spectrum results. Compared to MBGN, AMBGN exhibits a methylene peak at 2975 cm^−1^ and a characteristic amide group peak at 1572 cm^−1^. A marked N 1s response was observed for AMBGN following APTES modification, whereas the MBGN N 1s signal was weak (Fig. S5). Together with the reported zeta-potential change, these observations are consistent with successful APTES functionalization of MBGN.

**Fig. 2:**
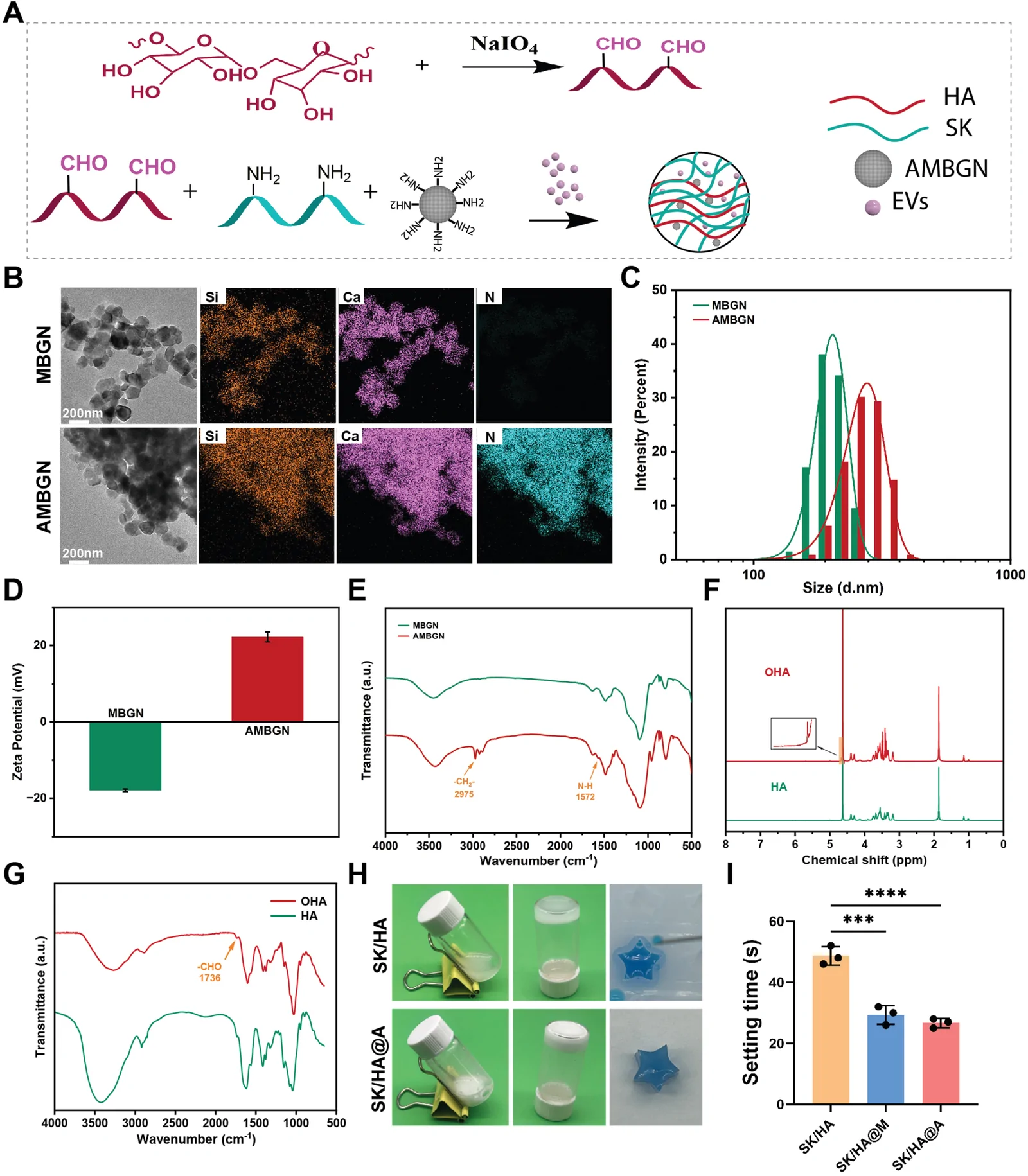
Preparation and physicochemical characterization of SK/HA@A hydrogel components. (A) Schematic illustration of the fabrication process, showing oxidation of HA to generate OHA, followed by Schiff base crosslinking with SK and AMBGN to form the hydrogel network. Tri-Exos can be subsequently incorporated. (B) TEM images and elemental mapping of MBGN and AMBGN, showing uniform Si and Ca distribution and successful surface amination confirmed by the presence of N. (C) Particle size distribution of MBGN and AMBGN measured by DLS. (D) Zeta potential of MBGN and AMBGN, confirming surface charge modification after amination. (E) FTIR spectra of MBGN and AMBGN. (F) 1HNMR spectra of HA and OHA, showing characteristic aldehyde peaks after oxidation. (G) FTIR spectra of HA and OHA, indicating successful formation of CHO groups. (H) Photographs of SK/HA and SK/HA@A pre-gel solutions demonstrating injectability and moldability. (I) Gelation time of SK/HA, SK/HA@M, and SK/HA@A hydrogels, showing accelerated crosslinking with incorporation of AMBGN. Data are presented as mean ± SD; ***P < 0.001, ****P < 0.0001.

The ^1^HNMR spectrum displayed a characteristic new peak at δ 4.6 ppm, indicative of aldehyde group formation (Fig. 2F). Further confirmation was obtained by FTIR analysis (Fig. 2G), which revealed a prominent CHO absorption peak at 1736 cm^−1^ and a C-H stretching peak at 2750 cm^−1^, corroborating the successful oxidation of HA. The formation of the SK/HA hydrogel and SK/HA@A hydrogel was visually confirmed: both systems exhibited high injectability and moldability, allowing them to be cast into various shapes (Fig. 2H). Incorporation of AMBGN significantly accelerated gelation, as measured by the gelation time assay, demonstrating that the addition of aminated nanoparticles enhances the crosslinking kinetics of the hydrogel network (Fig. 2I). These results collectively demonstrate that the SK/HA@A system provides a highly tunable, injectable, and mechanically robust scaffold suitable for subsequent exosome incorporation and bone tissue engineering applications.

The structural and mechanical properties of SK/HA-based hydrogels were systematically characterized. SEM imaging revealed that both SK/HA and SK/HA@A hydrogels exhibited highly porous 3D networks suitable for cell infiltration, and elemental mapping confirmed uniform distribution of C, O, and Si, indicating successful incorporation of AMBGN into the hydrogel matrix (Fig. 3A).

**Fig. 3:**
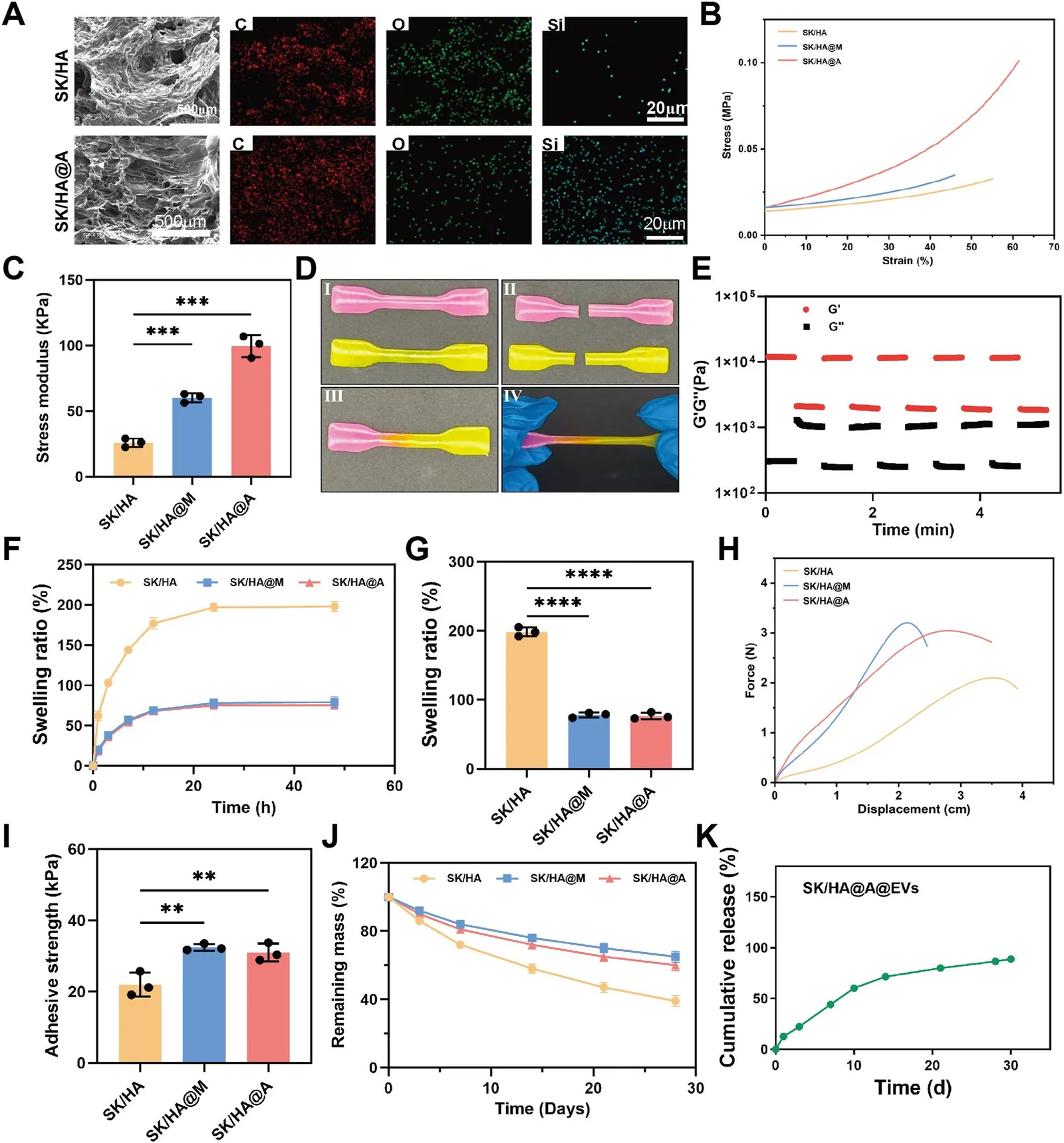
Physicochemical characterization and mechanical properties of SK/HA hydrogel variants. (A) SEM images and elemental mapping of SK/HA and SK/HA@A hydrogels showing the 3D porous network structure and uniform distribution of carbon (C), oxygen (O), and silicon (Si). (B) Stress-strain curves of SK/HA, SK/HA@M, and SK/HA@A hydrogels under compression. (C) Compressive modulus of hydrogel variants, showing significant enhancement upon incorporation of AMBGN. (D) Photographs demonstrating injectability and self-healing behavior of SK/HA@A hydrogels: self-healing demonstration with hydrogel cut and rejoined. (E) Oscillatory rheology showing storage (G′) and loss (G″) moduli of SK/HA@A hydrogel. (F–G) Swelling ratio of hydrogels over time, indicating reduced swelling upon AMBGN incorporation. (H) Compression force-displacement curves showing mechanical strength. (I) Adhesive strength of hydrogel variants. (J) In vitro degradation profile measured by remaining mass over 28 days. (K) Cumulative release of exosomes from SK/HA@A@EVs hydrogel over 30 days. Data are presented as mean ± SD; **P < 0.01, ***P < 0.001, ****P < 0.0001. (For interpretation of the references to color in this figure legend, the reader is referred to the Web version of this article.)

Mechanical evaluation demonstrated that incorporation of AMBGN substantially enhanced hydrogel stiffness. Stress-strain measurements showed that SK/HA@A displayed higher compressive strength compared to SK/HA and SK/HA@M (Fig. 3B and C). To further evaluate the mechanical stability of the hydrogels under repeated loading, cyclic compression tests were performed on SK/HA, SK/HA@M, and SK/HA@A for 20 consecutive cycles up to 20% strain (Fig. S6). All three formulations exhibited broadly similar stress–strain profiles during the first cycle, intermediate cycles (cycles 2–19), and the 20th cycle. Although a modest decrease in stress response was observed by the 20th cycle, no obvious abrupt loss of load-bearing capacity occurred. Among the three formulations, SK/HA@A exhibited the highest compressive stress, followed by SK/HA@M and SK/HA, indicating differences in their compressive mechanical responses. Overall, these results demonstrate that all three hydrogels maintained their compressive response during the tested loading cycles, supporting their short-term cyclic compressive stability. Photographs demonstrated injectability and robust self-healing behavior, where hydrogels could recover their structure after cutting (Fig. 3D). Oscillatory rheology further confirmed the viscoelastic properties of SK/HA@A, with storage modulus (G′) consistently higher than loss modulus (G″), indicating a predominantly elastic behavior (Fig. 3E).

Swelling tests revealed that AMBGN incorporation reduced hydrogel swelling compared to SK/HA alone, stabilizing the hydrogel network for sustained function (Fig. 3F and G). Adhesive strength measurements demonstrated that SK/HA@A had superior mechanical robustness and tissue adhesion compared with other formulations (Fig. 3H and I). In vitro degradation assays showed gradual hydrogel mass loss over 28 days, with SK/HA@A displaying a slower degradation profile, suitable for long-term therapeutic delivery (Fig. 3J). Cumulative exosome release studies indicated that SK/HA@A@EVs hydrogels released exosomes steadily over 30 days, demonstrating their potential as a sustained delivery platform for bioactive cargo (Fig. 3K).

These results collectively establish that the incorporation of AMBGN into SK/HA hydrogels significantly improves mechanical properties, swelling stability, and sustained exosome release, supporting their suitability for bone tissue engineering applications.

### 3.3 SK/HA@A@EVs Promote Proliferation, osteogenic differentiation, angiogenesis, and Molecular Regulation in BMSCs and HUVECs

The therapeutic effects of the SK/HA@A@EVs system were further examined through the comprehensive assessment of BMSCs and HUVECs to evaluate proliferation, viability, osteogenic differentiation, migratory capacity, gene regulation, autophagic activity, and oxidative stress responses. As shown in Fig. 4A and B, compared with the control and other hydrogel groups, the SK/HA@A@EVs group exhibited significantly enhanced proliferation of both BMSCs and HUVECs, as indicated by CCK-8 assay. This proliferation-promoting effect was further supported by live/dead staining (Fig. 4C), which revealed a markedly greater proportion of viable cells in the SK/HA@A@EVs group. These results indicate a favorable cellular response to the hydrogel system, likely contributing to enhanced regenerative capacity. SK/HA@A@EVs treatment markedly increased ALP activity, as evidenced by intense ALP staining (Fig. 4D), indicating early osteogenic induction. Subsequent alizarin red S staining after 21 days of induction revealed substantially increased calcium deposition (Fig. 4E), confirming the strong promotion of matrix mineralization and late-stage osteogenic differentiation in BMSCs.

**Fig. 4:**
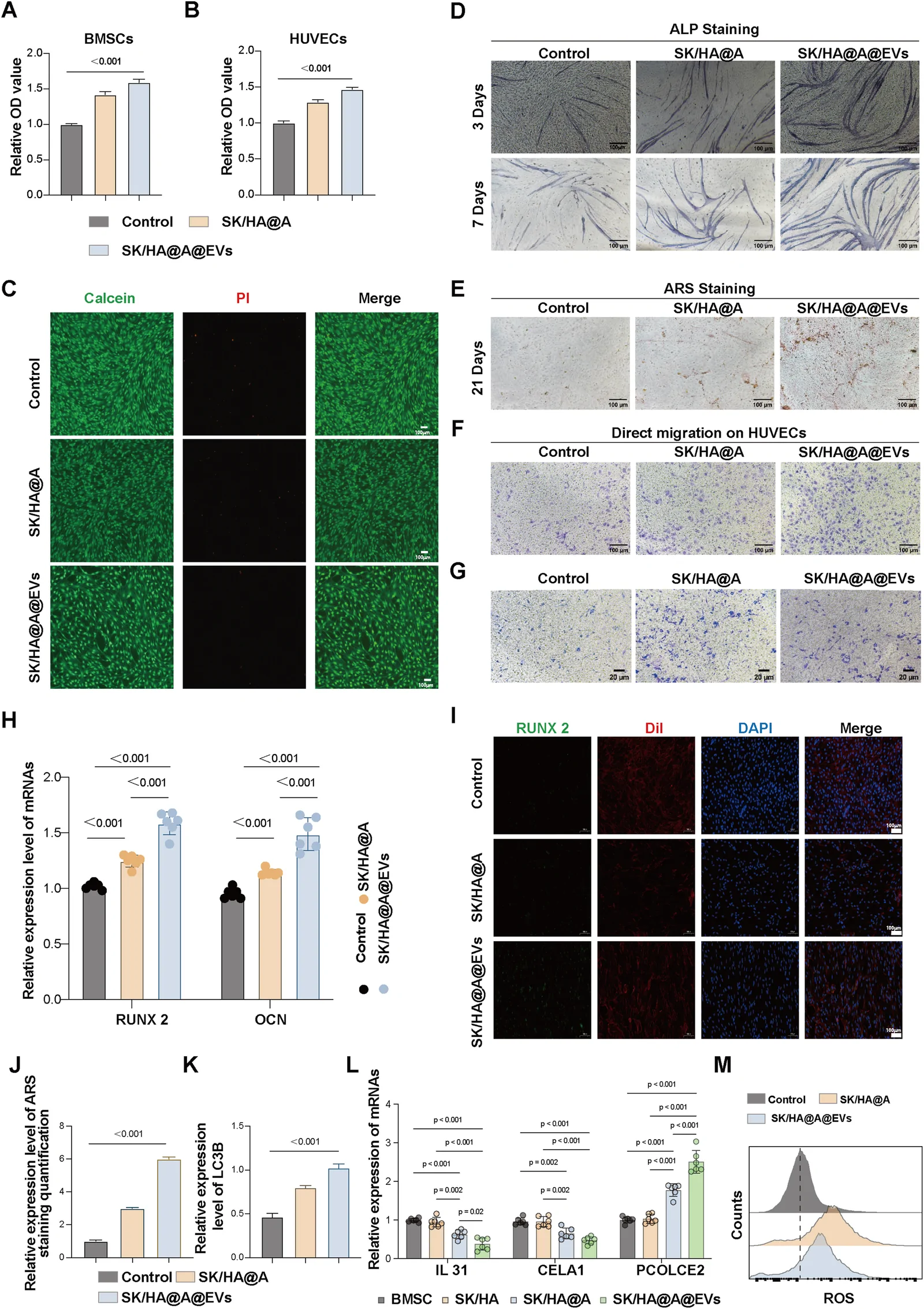
SK/HA@A@EVs Promote Proliferation, Osteogenic Differentiation, Angiogenesis, and Molecular Regulation in BMSCs and HUVECs (A–B) CCK-8 assays showing that compared with the control and other hydrogels, SK/HA@A@EVs significantly enhanced the proliferation of bone marrow mesenchymal stem cells (BMSCs) and human umbilical vein endothelial cells (HUVECs). (C) Live/dead staining revealed a markedly increased proportion of viable cells in the SK/HA@A@EVs group, confirming superior cell survival. (D) Alkaline phosphatase (ALP) staining after 7 days of osteogenic induction revealed strong ALP activity in the BMSCs treated with SK/HA@A@EVs, indicating enhanced early-stage osteogenic differentiation. (E) Alizarin red S (ARS) staining on day 21 revealed significantly increased mineralized nodule formation in the SK/HA@A@EVs group, confirming robust matrix mineralization and late-stage osteogenesis. (F–G) Wound healing assays revealed that SK/HA@A@EVs significantly improved HUVEC migration, supporting its angiogenic potential. (H) RT-qPCR analysis revealed upregulated expression of the osteogenic markers RUNX2 and OCN in the BMSCs treated with SK/HA@A@EVs. (I) Immunofluorescence staining revealed enhanced nuclear expression of RUNX2 in SK/HA@A@EVs-treated BMSCs, further confirming osteogenic transcriptional activation. (J–K) RT-qPCR analysis revealed upregulation of the expression of the autophagy-related genes ARS and LC3B, indicating the activation of autophagic pathways that support cellular homeostasis and osteogenesis. (L) Gene expression profiling of osteoporosis-associated targets revealed that SK/HA@A@EVs significantly downregulated the pro-osteoporotic genes IL31 and CELA1 while upregulating the protective gene PCOLCE2, suggesting targeted and favorable gene regulation. (M) Intracellular ROS levels measured by flow cytometry indicated that SK/HA@A@EVs initially increased oxidative stress in BMSCs, but coculture with BMSCs at a higher dose (200 mg) significantly reduced ROS accumulation, reflecting potential redox homeostasis restoration. (For interpretation of the references to color in this figure legend, the reader is referred to the Web version of this article.)

To assess angiogenic potential, wound healing assays were performed. As shown in Fig. 4F and G, SK/HA@A@EVs significantly enhanced the migration of HUVECs, suggesting a positive effect on endothelial mobility and potential vascularization at the site of bone injury. Moreover, RT-qPCR analysis of osteogenic gene expression (Fig. 4H) revealed significantly upregulated RUNX2 and OCN mRNA levels in SK/HA@A@EVs-treated BMSCs, indicating the activation of key transcriptional pathways governing osteogenesis. This finding was visually confirmed by immunofluorescence staining (Fig. 4I), which revealed increased nuclear expression of RUNX2 in the treated cells. The effect of the SK/HA@A@EVs system on autophagy was also evaluated.

RT-qPCR revealed increased expression of autophagy-related genes, including ARS and LC3B (Fig. 4J and K), suggesting that SK/HA@A@EVs may facilitate cellular homeostasis and support osteogenic function via the activation of autophagic pathways. Gene expression modulation by the hydrogel system was further examined. As shown in Fig. 4L, qPCR analysis demonstrated that SK/HA@A significantly suppressed the expression of IL31 and CELA1, whereas SK/HA@A@EVs further enhanced this suppressive effect. Conversely, PCOLCE2 expression was upregulated in the SK/HA@A group but further increased with SK/HA@A@EVs treatment. These findings suggest that the SK/HA@A@EVs hydrogel not only promotes bone formation but also preferentially regulates key osteoporosis-associated genes, which is in agreement with previous colocalization and functional validation results (Fig. 2). The dual regulation—suppressing pro-osteoporotic genes while promoting protective genes—emphasizes the precise molecular modulation conferred by this system. Flow cytometry revealing elevated levels of ROS in BMSCs response to SK/HA@A@EVs alone, indicating a stress response (Fig. 4M). However, when the SK/HA@A@EVs system was cocultured with BMSCs at a relatively high concentration (200 mg), ROS accumulation was significantly reduced, indicating that the hydrogel at this dosage may help restore redox homeostasis, potentially through feedback regulation by BMSCs.

In summary, these results demonstrate that SK/HA@A@EVs significantly increase BMSC and HUVEC proliferation, survival, osteogenic differentiation, angiogenic migration, autophagy, and favorable gene modulation while also helping regulate oxidative stress. The SK/HA@A@EVs system demonstrates diverse therapeutic actions, establishing its potential as a prospective bioactive scaffold for bone tissue engineering and osteoporosis management.

### 3.4 SK/HA@A@EVs Promote Osteogenesis and Angiogenesis in BMSCs via autophagy activation and enhanced cell survival

To comprehensively evaluate the biological effects of SK/HA@A@EVs on BMSCs, we explored the effects on osteogenic differentiation, autophagy, and cell survival signaling through a combination of immunofluorescence, staining assays, Western blotting (WB), and gene expression analysis. Immunofluorescence staining for VEGF and actin (Fig. 5A) revealed that VEGF expression was markedly elevated in the SK/HA@A@EVs group, indicating enhanced proangiogenic activity and cytoskeletal remodeling in the BMSCs. These findings indicate a conducive microenvironment for osteogenesis and tissue regeneration. Alizarin red S staining corroborated osteogenic differentiation, showing a dose-dependent increase in calcium deposition in BMSCs with increasing SK/HA@A@EVs concentration (Fig. 5B). These results were consistent with the significantly elevated alkaline phosphatase (ALP) activity (Fig. 5C), confirming that SK/HA@A@EVs robustly enhance both early- and late-stage osteogenesis.

**Fig. 5:**
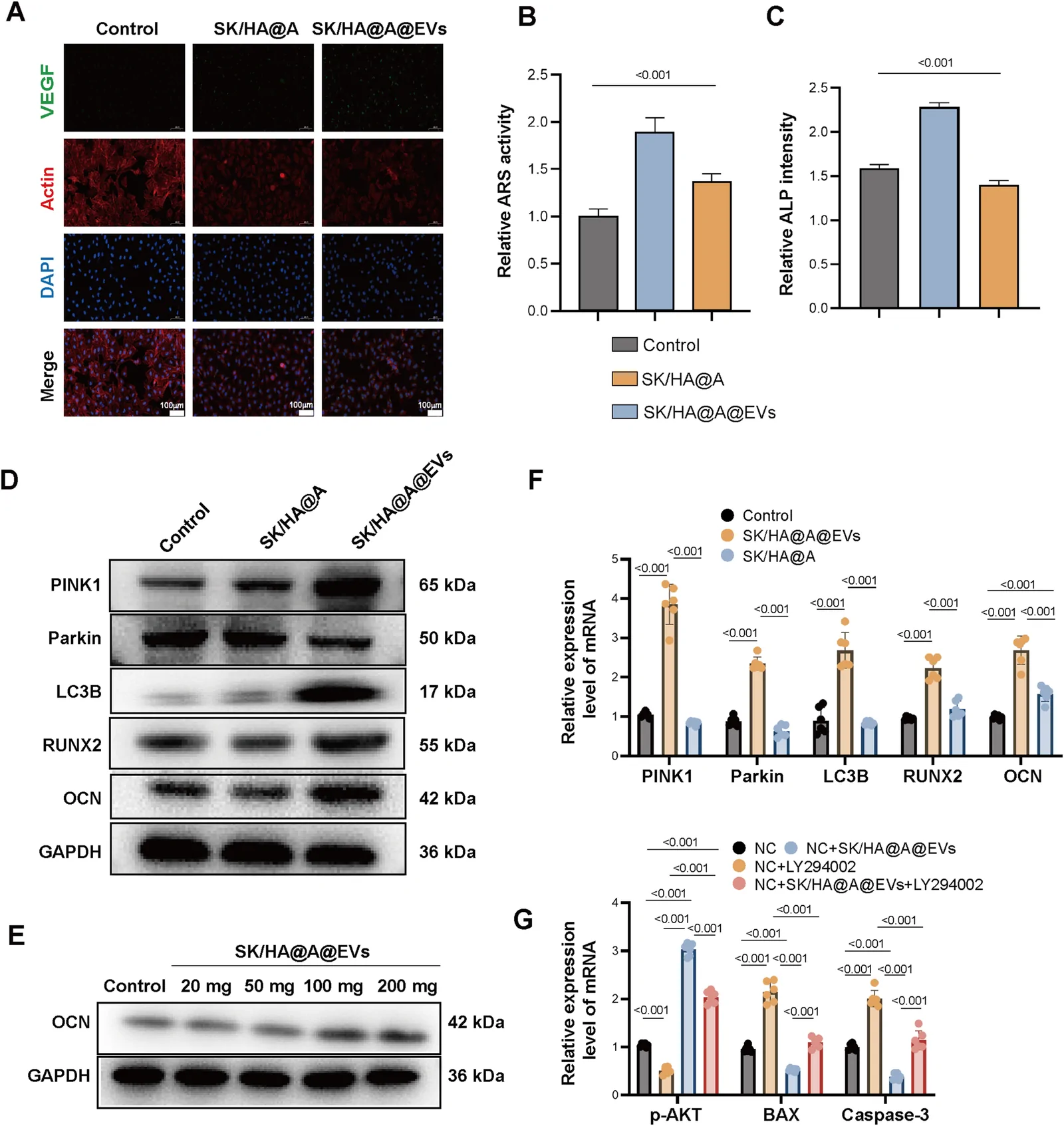
SK/HA@A@EVs Promote Osteogenesis and Angiogenesis in BMSCs by Enhancing Autophagy and Cell Survival (A) Immunofluorescence staining for VEGF and actin in BMSCs revealed increased VEGF expression and cytoskeletal remodeling in the SK/HA@A@EVs group, indicating enhanced proangiogenic signaling and structural support for osteogenesis. (B) Alizarin red S (ARS) staining revealed a dose-dependent increase in calcium deposition following SK/HA@A@EVs treatment, confirming enhanced matrix mineralization and late-stage osteogenesis. (C) Alkaline phosphatase (ALP) activity assays demonstrated significantly elevated enzymatic activity in SK/HA@A@EVs-treated BMSCs, indicating early osteogenic differentiation. (D–E) Western blot analysis revealed the upregulation of osteogenic markers (RUNX2 and OCN) and autophagy-related proteins (PINK1, Parkin, and LC3B) in SK/HA@A@EVs-treated cells, suggesting the concurrent activation of osteogenesis and mitophagy. (F) RT-qPCR analysis confirmed the significant transcriptional upregulation of osteogenic genes (RUNX2 and OCN) and autophagy-related genes (PINK1, Parkin, and LC3B) in BMSCs treated with SK/HA@A@EVs. (G) Western blot and RT-qPCR analyses of cell survival markers revealed increased expression and phosphorylation of AKT (p-AKT) and reduced expression of the pro-apoptotic genes BAX and caspase-3, indicating enhanced cell survival signaling and apoptosis inhibition. (For interpretation of the references to color in this figure legend, the reader is referred to the Web version of this article.)

Western blot analysis provided deeper insights into the underlying molecular mechanisms (Fig. 5D and E). Key proteins associated with osteogenic differentiation (such as RUNX2 and OCN) and autophagy (including PINK1, Parkin, and LC3B) were found to be upregulated following SK/HA@A@EVs treatment. These findings indicate the activation of mitochondrial quality control and autophagic pathways, which are known to support osteoblastic function and metabolic adaptation. To validate these observations at the transcriptional level, RT-qPCR analysis was conducted. As shown in Fig. 5F, genes involved in osteogenesis (RUNX2 and OCN) and autophagy (PINK1, Parkin, and LC3B) were significantly upregulated in response to SK/HA@A@EVs. These results confirm that the hydrogel system facilitates both matrix formation and intracellular turnover, processes that are essential for effective bone regeneration. Additionally, we examined cell survival signaling. RT-qPCR and Western blot analyses demonstrated that SK/HA@A@EVs upregulated both total and phosphorylated AKT—an essential prosurvival kinase—while simultaneously downregulating the pro-apoptotic proteins BAX and caspase-3 (Fig. 5G), indicating that SK/HA@EVs have a protective effect against apoptosis that preserves BMSC viability under stress and during osteogenic differentiation. Overall, SK/HA@A@EVs not only drive dose-dependent osteogenic maturation and increase autophagy-related gene and protein expression but also improve BMSC survival via AKT pathway activation and apoptosis inhibition, underscoring their promise as an intelligent biomaterial for bone regeneration and osteoporosis treatment [35].

### 3.5 SK/HA@A@EVs facilitate bone regeneration and Angiogenesis In Vivo in a femoral defect model

To validate the regenerative potential of SK/HA@A@EVs in vivo, we established a femoral defect model and evaluated bone formation and angiogenesis using micro-CT imaging, histological staining, and molecular analysis. All animal experiments were approved by the Animal Research Ethics Committee of Shanghai Sixth People's Hospital and Shanghai Rat&Mouse Biotech Co.,Ltd (Approval no. 20220915 (3)), and animal experiments were conducted according to the National Institutes of Health Guidelines for the Care and Use of Laboratory Animals. The animals were assigned to three groups: control, SK/HA@A, and SK/HA@A@EVs. Three-dimensional reconstructions and cross-sectional images from micro-CT analyses (Fig. 6A) revealed markedly enhanced bone regeneration in the SK/HA@A@EVs group compared with the control and SK/HA@A groups. Quantitative assessment of bone mineral density (BMD) (Fig. 6B) further confirmed a significant increase in mineral content in the SK/HA@A@EVs group, suggesting accelerated bone remodeling. Immunohistochemical staining for osteopontin (OPN), a key osteogenic marker, revealed stronger OPN expression in femoral sections from SK/HA@A@EVs-treated animals (Fig. 6C), indicating elevated osteoblastic activity and enhanced bone matrix deposition. To evaluate angiogenesis, CD31 immunostaining was performed (Fig. 6D). The SK/HA@A@EVs group displayed elevated CD31-positive vascularization relative to the control and SK/HA@A groups, demonstrating pronounced neovascularization at the defect site.

**Fig. 6:**
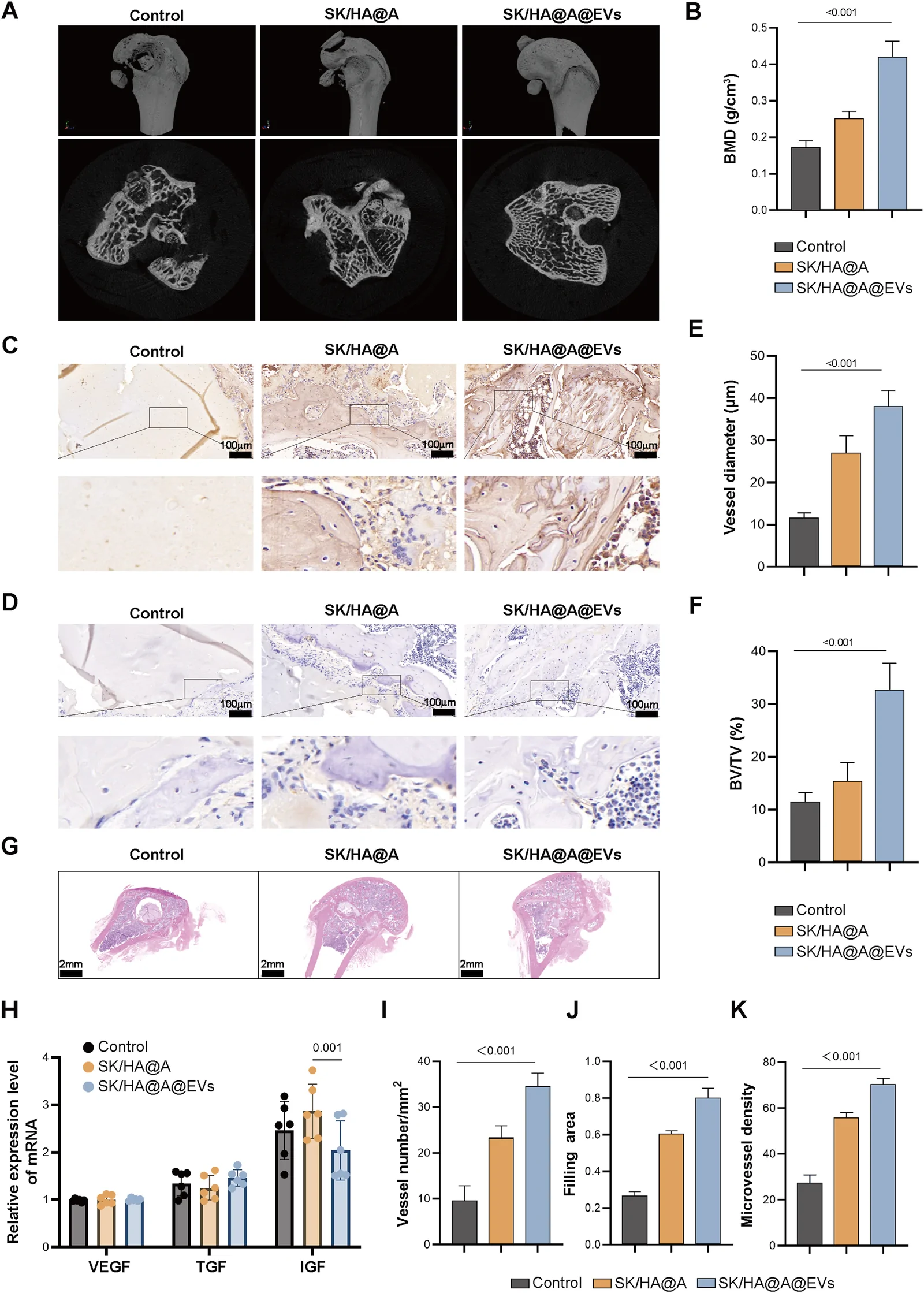
SK/HA@A@EVs Promote Bone Regeneration and Angiogenesis In Vivo (A) Representative 3D micro-CT reconstructions and cross-sectional images of femoral defects showing markedly enhanced bone formation in the SK/HA@A@EVs group compared with the control and SK/HA@A groups. (B) Quantitative analysis of bone mineral density (BMD) revealed significantly increased mineral content in the SK/HA@A@EVs group, indicating accelerated bone regeneration. (C) Immunohistochemical staining for osteopontin (OPN) demonstrated increased OPN expression in SK/HA@A@EVs-treated femoral tissue, reflecting enhanced osteoblastic activity and bone matrix deposition. (D) CD31 immunostaining of defect regions revealed a greater number of CD31-positive vessels in the SK/HA@A@EVs group, indicating robust neovascularization. (E) Vessel diameter and (I) vessel density per mm^3^ were significantly increased in SK/HA@A@EVs-treated animals, supporting improved vascular network formation. (F) Quantitative histomorphometric analysis of the bone volume/total volume ratio (BV/TV) revealed a significant increase in the SK/HA@A@EVs group, which was consistent with enhanced structural bone regeneration. (G) H&E staining confirmed superior tissue architecture and new bone formation in the SK/HA@A@EVs group compared with the other groups. (H) RT-qPCR analysis of osteogenic and angiogenic gene expression (VEGF, TGF-β, and IGF) in femoral tissues revealed significant upregulation in the SK/HA@A@EVs group, suggesting coordinated activation of bone and vascular repair pathways. (J–K) Morphometric assessment revealed significantly larger vessel filling areas and higher microvessel density in the SK/HA@A@EVs-treated defects, confirming enhanced vascularization.

Further quantification revealed that the vessel diameter (Fig. 6E) and vessel density per mm^3^ (Fig. 6I) were significantly increased, indicating improved vascular connectivity and microcirculation. The SK/HA@A@EVs group demonstrated a markedly increased bone volume-to-total volume ratio (BV/TV), a pivotal indicator of bone regeneration, as corroborated by imaging and histological analyses (Fig. 6F). Femoral tissue H&E staining confirmed the enhanced tissue architecture and bone formation in the SK/HA@A@EVs cohort (Fig. 6G). To explore the underlying molecular mechanisms, RT-qPCR analysis of femoral tissue was conducted (Fig. 6H). Compared with the control group, the SK/HA@A@EVs group showed markedly increased expression of pivotal osteogenic and angiogenic factors—VEGF, TGF-β, and IGF— indicating synergistic stimulation of both bone formation and vascular development pathways. Additionally, morphometric analysis revealed a greater vessel filling area (Fig. 6J) and microvessel density (Fig. 6K) in SK/HA@A@EVs-treated tissues, confirming enhanced vascularization within the regenerative microenvironment. Collectively, these results demonstrate that SK/HA@A@EVs significantly enhance bone regeneration and angiogenesis in vivo. This is evidenced by increased BMD, BV/TV, and neovascularization, as well as the upregulation of key osteogenic and angiogenic genes. These findings highlight the therapeutic potential of SK/HA@A@EVs in promoting coupled bone and vascular repair in bone defect models.

## 4 Discussion

Bone defects and impaired regeneration remain a significant challenge in regenerative medicine, particularly for extensive lesions caused by trauma, infection, tumor resection, or degenerative bone diseases [39]. Repair failure rates in such conditions can reach 15-20%, resulting in impaired physiological function, prolonged patient suffering, and increased healthcare costs [40]. Conventional treatments, including autologous or allogeneic bone grafts and synthetic bone substitutes, provide some benefit [41]; however, donor site morbidity, immune rejection, and postoperative complications limit their clinical efficacy [42]. Therefore, innovative strategies are urgently needed to overcome these limitations and enhance the success of bone defect repair.

Bone tissue engineering strategies based on nucleic acid therapeutics offer a promising approach. Osteoporotic and degenerative conditions are often characterized by dysregulated bone metabolism, excessive matrix degradation, and imbalanced osteoclast/osteoblast activity. In this context, exosomes engineered with siRNA and mRNA have been developed to protect bone matrix integrity and promote regeneration. Despite their bioactive cargo, extracellular vesicles remain vulnerable in complex biological environments. Biomaterials, particularly hydrogels, have been employed as delivery platforms due to their biomimetic properties, biocompatibility, and suitability as 2D and 3D scaffolds for cell attachment, proliferation, and differentiation [43], [44]. Hydrogels mimic the extracellular matrix and can be loaded with growth factors or cells to facilitate bone regeneration [45]; however, they often lack the ability to maintain biosignaling and activate key regenerative pathways [46].

To address these challenges, we developed the SK/HA@A@EVs system, combining a light-responsive, highly porous SK/HA@A hydrogel with exosomes derived from iPSC-MSCs [47]. This integrated platform generates a bioactive microenvironment that robustly promotes osteogenesis and angiogenesis [23].

Mechanistically, SK/HA@A@EVs enhanced BMSC proliferation and osteogenic differentiation through sustained delivery of bioactive factors, including growth factors, cytokines, and noncoding RNAs [23], [48]. Sustained release prolonged exosomal activity, resulting in increased ALP activity, calcium deposition, and upregulation of osteogenic markers (COL1, ALP, RUNX2) [49], [50]. Simultaneously, the system promoted angiogenic responses via BMSC-mediated signaling, enhancing migration and capillary-like structure formation [51]. In femoral defect models, SK/HA@A@EVs outperformed controls, leading to higher bone density (micro-CT) and trabecular maturation (histology), along with elevated OCN and BMP-2 expression [23]. Microvascular density was also increased, confirming the dual osteogenic-angiogenic effect. Importantly, the SK/HA@A@EVs system activated PINK1/Parkin-mediated mitophagy [52], [53], and PI3K/Akt inhibition (LY294002) attenuated this effect, demonstrating that this pathway is critical for enhancing mitochondrial function and promoting osteogenesis [54]. Additionally, the system promoted BMSC autophagy and cell survival, further supporting osteogenesis and angiogenesis and providing a molecular basis for coordinated bone regeneration. Collectively, these results highlight the potential of SK/HA@A@EVs as a multifunctional biomaterial for clinical bone defect repair, integrating cellular proliferation, differentiation, vascularization, and molecular mechanisms.

Bioinformatic analyses identified key regulators of mitophagy and osteogenesis in the SK/HA@A@EVs system [55], [56], [57]. Subsequent in vitro studies confirmed the pivotal roles of these factors: overexpression recapitulated the regenerative effects of the hydrogel-exosome platform, whereas knockdown attenuated them [58], [59]. These findings elucidate the underlying regenerative mechanisms and highlight the potential for targeted bone therapies. Furthermore, bioinformatics-guided gene modulation offers a promising avenue for precise intervention in genetic bone disorders [60], [61], [62], [63], [64], [65], [66].

Despite these encouraging results, further validation in larger animal models, such as nonhuman primates, is necessary to better predict clinical outcomes in humans. Although the SK/HA@A@EVs system demonstrates robust osteogenic and angiogenic potential, additional studies are required to evaluate long-term bone remodeling, hydrogel–exosome interactions under physiological conditions, and the involvement of key molecular pathways in vivo. By integrating porous hydrogels with iMSC-derived exosomes, the SK/HA@A@EVs platform enhances bone repair through mitophagy-mediated osteogenesis and angiogenesis. While further preclinical validation is warranted, this approach presents significant promise for clinical bone defect repair. Future studies will focus on optimizing system performance and exploring novel therapeutic targets for broader regenerative applications.

## 5 Conclusion

In this study, potential targets for pro-osteoporotic bone regeneration were identified through integrative proteomic and genomic analyses, and a SK/HA@A@EVs hydrogel delivery system combining nucleic acid therapeutics with functionalized exosomes was developed. This system integrates a highly porous hydrogel scaffold with engineered exosomes to enhance both osteogenic and angiogenic potential, representing a novel strategy for bone defect repair. Our findings demonstrate that the SK/HA@A@EVs platform effectively promotes osteogenic differentiation, mitophagy-mediated mitochondrial regulation, and angiogenesis, all of which are essential for robust bone regeneration. Furthermore, this study provides mechanistic insights by linking the biological activity of exosome therapy with the synergistic mechanical and biochemical effects of the hydrogel matrix. Overall, the SK/HA@A@EVs system offers a promising strategy for bone tissue engineering and holds potential for clinical translation in the repair of bone defects.

## CRediT authorship contribution statement

**Ning Xu:** Writing – original draft, Investigation, Formal analysis, Data curation. **Chenjun Liu:** Writing – original draft, Methodology, Investigation. **Jieyuan Zhang:** Methodology, Formal analysis. **Huiyong Dai:** Methodology, Formal analysis. **Shengting Wu:** Methodology. **Sa Song:** Methodology. **Junjie Guan:** Writing – review & editing, Supervision. **Xin Qi:** Writing – review & editing. **Wei Zhang:** Supervision, Funding acquisition.

## Declaration of competing interest

The authors declare that they have no known competing financial interests or personal relationships that could have appeared to influence the work reported in this paper.

## Figures and Tables

**figs1:**
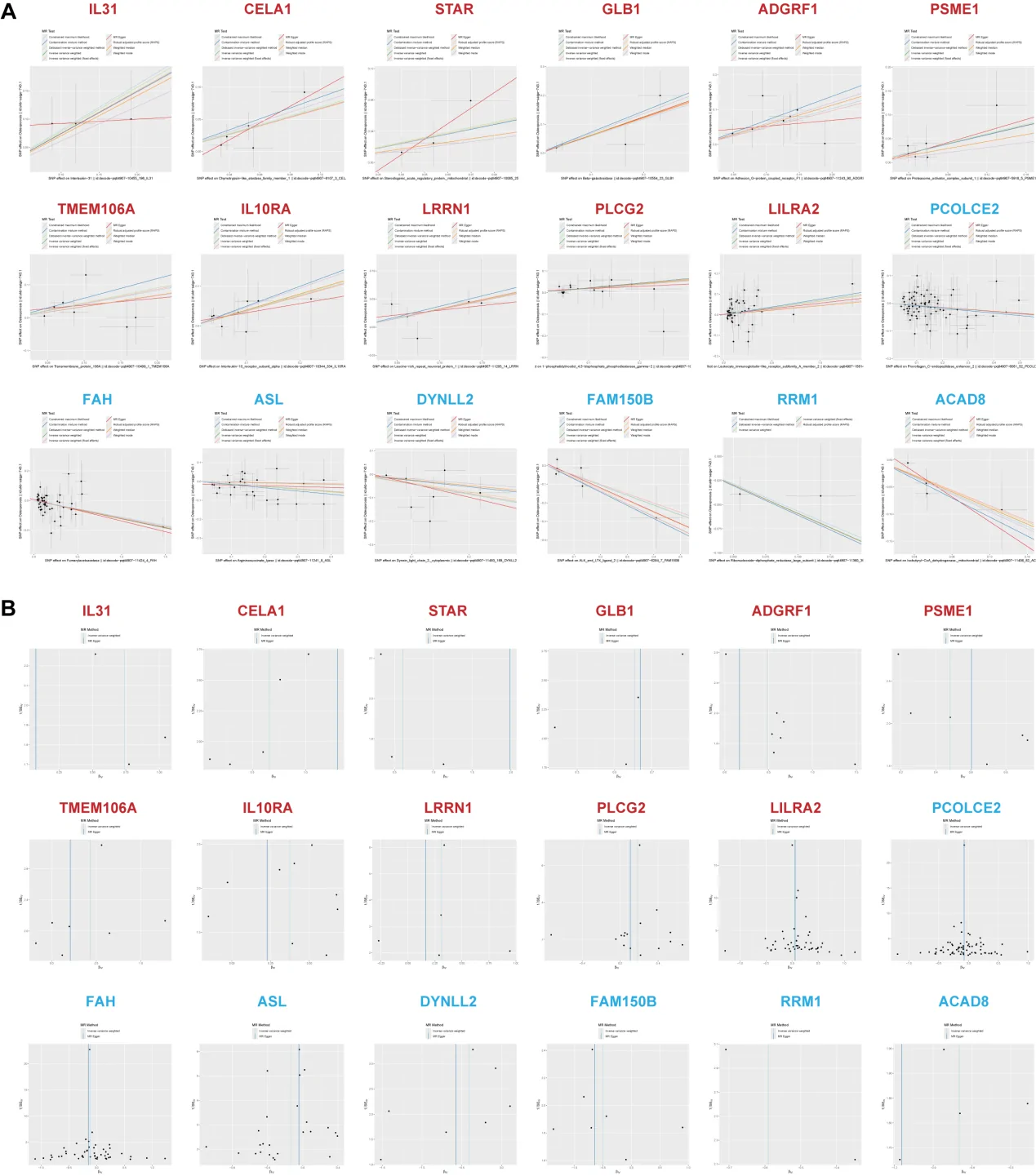


**figs2:**
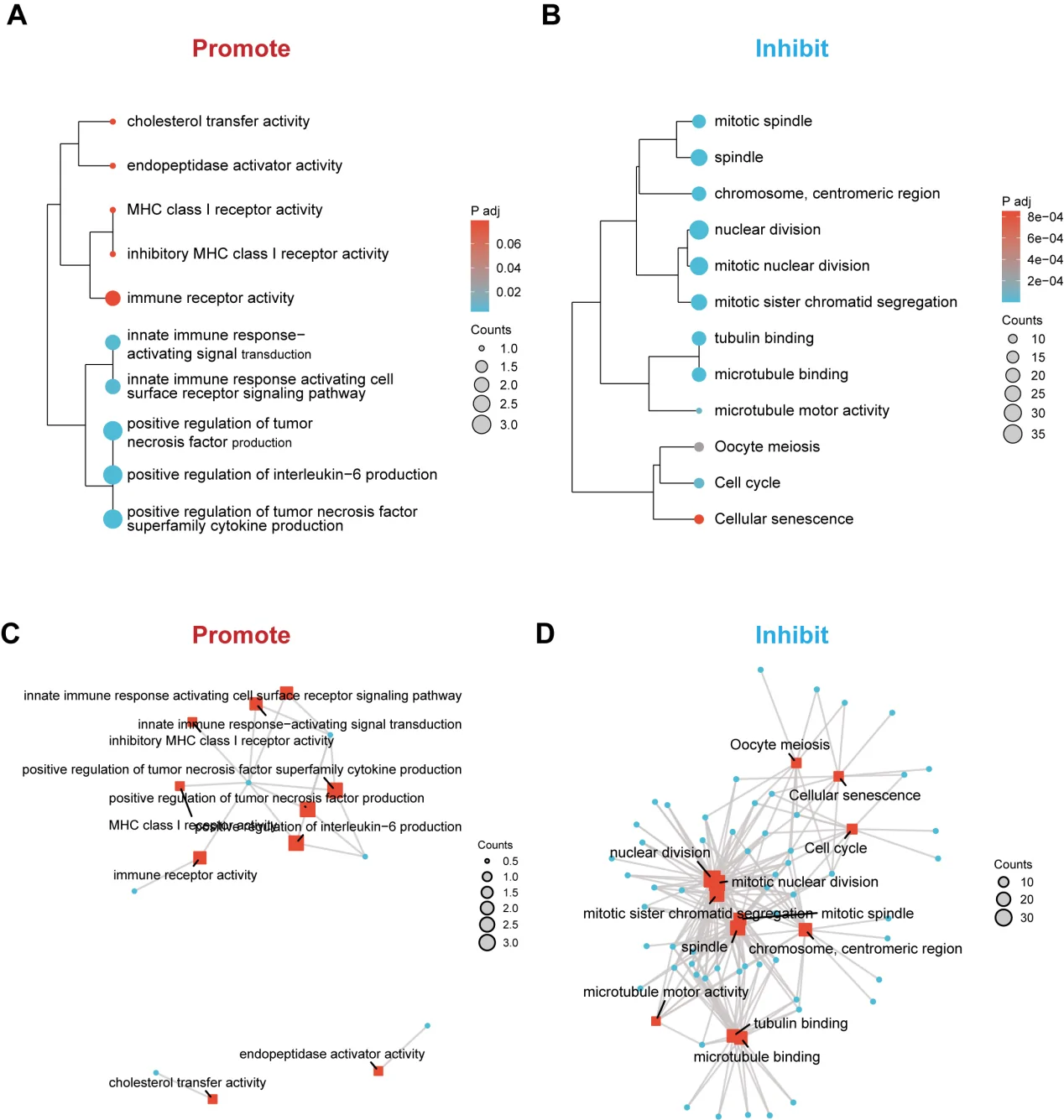


**figs3:**
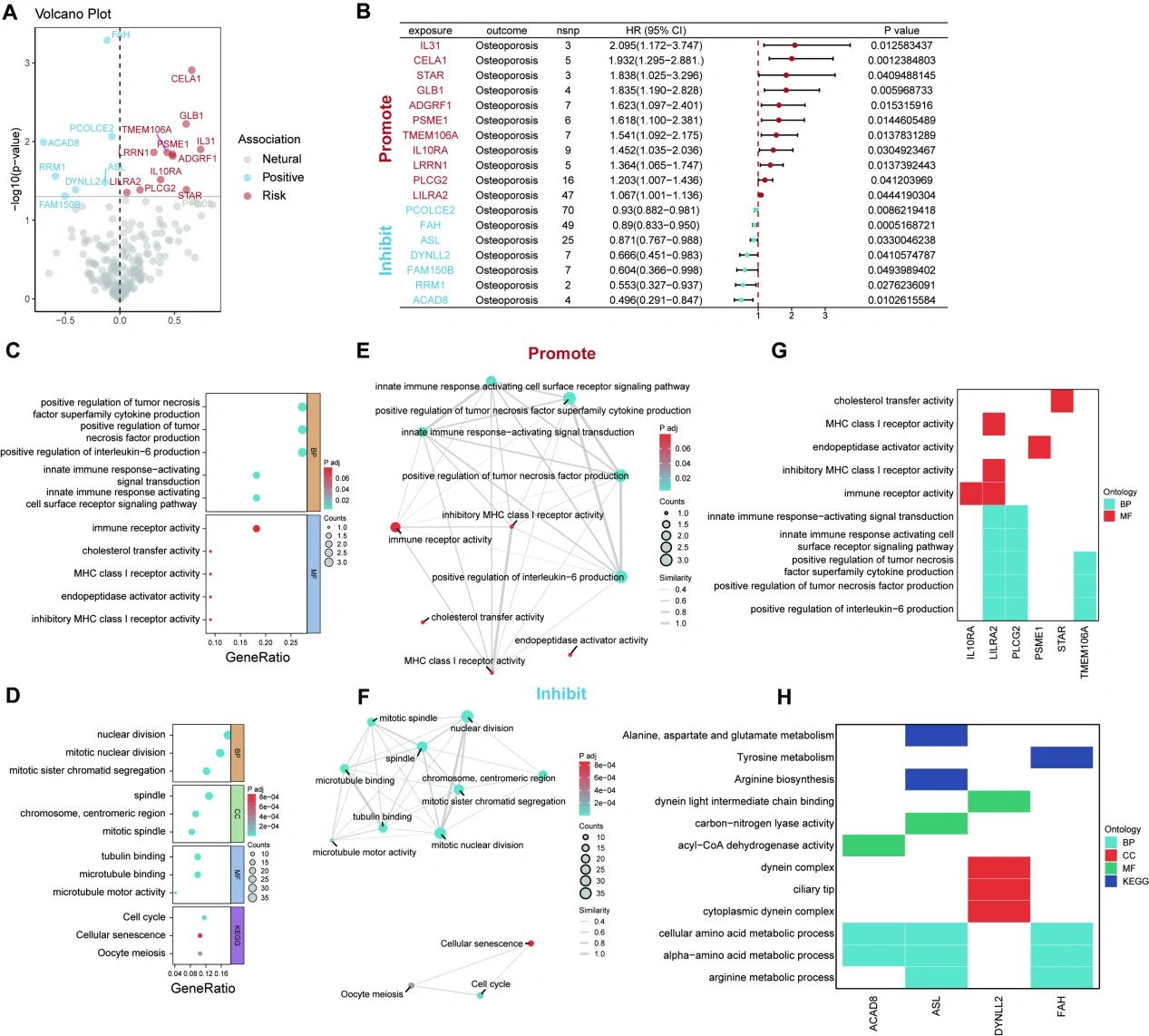


**figs4:**
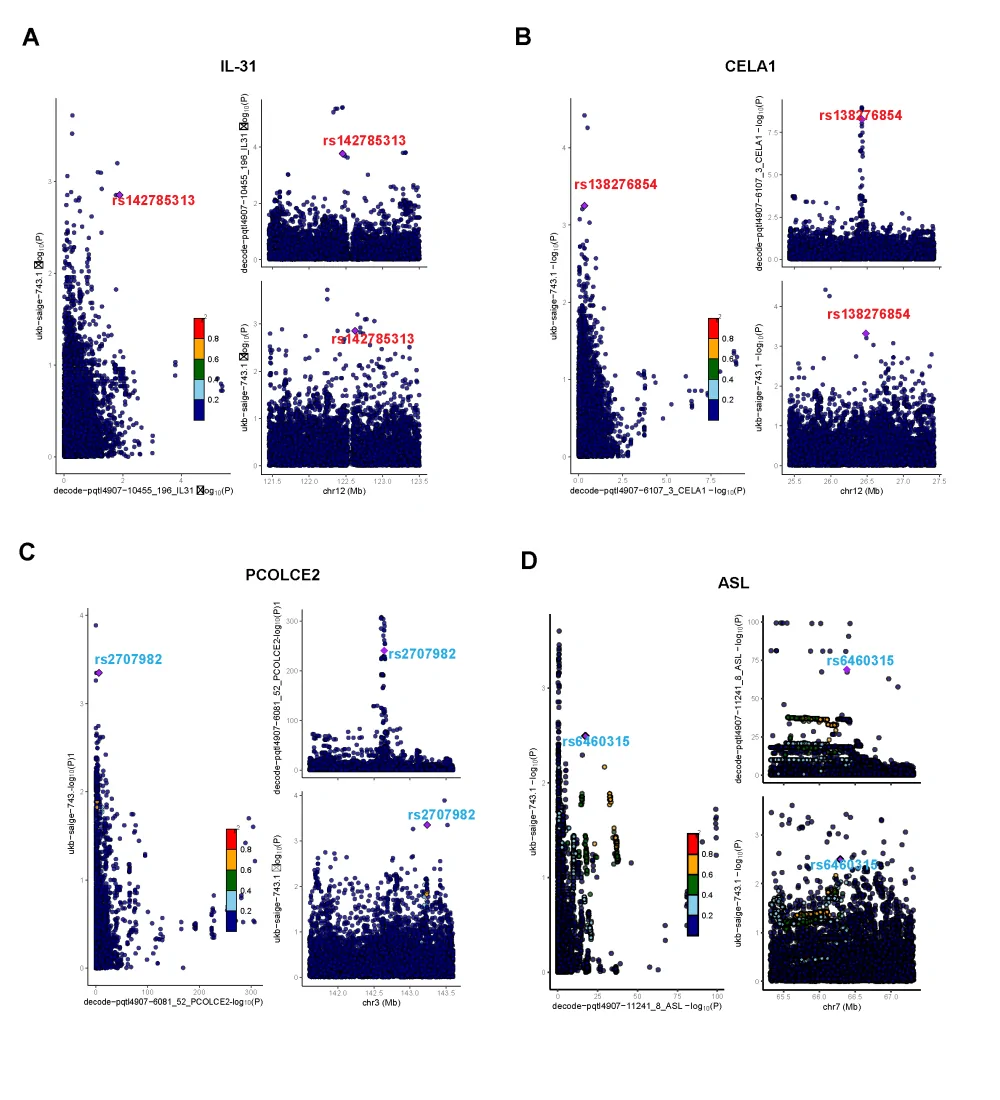


**figs5:**
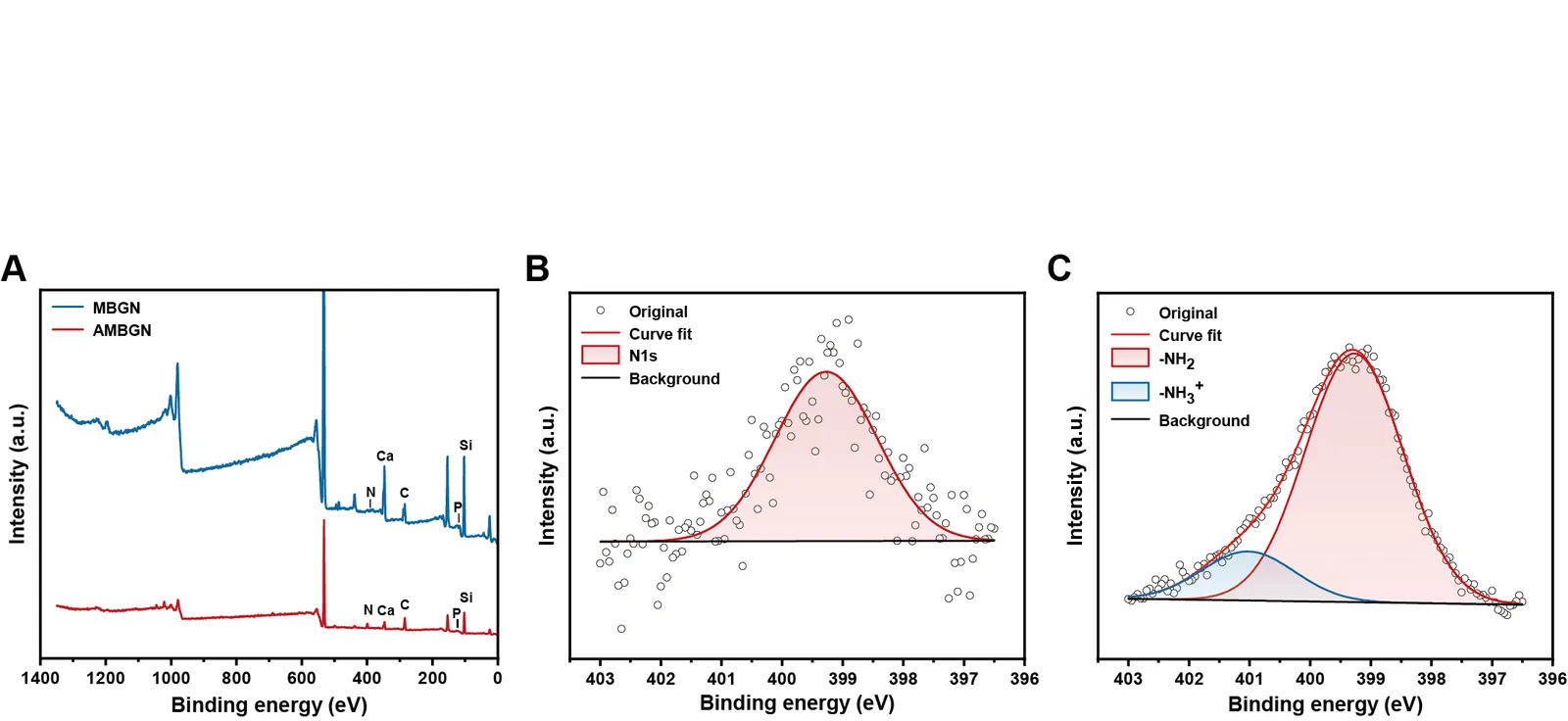


**figs6:**
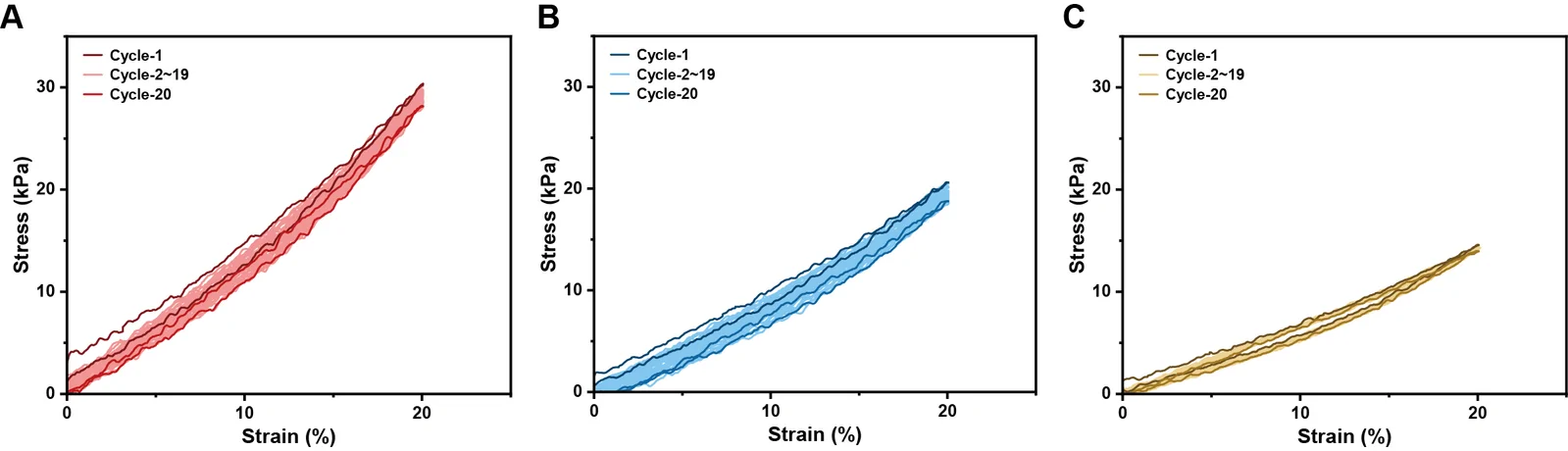


## Data Availability

The data that support the findings of this study are available from the corresponding author upon reasonable request.
